# Recent advances in understanding and managing rosacea

**DOI:** 10.12688/f1000research.16537.1

**Published:** 2018-12-03

**Authors:** Joerg Buddenkotte, Martin Steinhoff

**Affiliations:** 1 Department of Dermatology and Venereology, Hamad Medical Corporation, Doha, Qatar; 2 Translational Research Institute, Hamad Medical Corporation, Doha, Qatar; 3 Weill Cornell Medicine-Qatar, Doha, Qatar; 4 Medical School, Qatar University, Doha, Qatar; 5 Weill Cornell Medicine, New York, NY, USA

**Keywords:** Rosacea, Classification, Pathophysiology, Inflammation, Cathelicidin, Immunity, Therapy

## Abstract

Rosacea is a common chronic inflammatory skin disease of the central facial skin and is of unknown origin. Currently, two classifications of rosacea exist that are based on either “preformed” clinical subtypes (erythematotelangiectatic, papulopustular, phymatous, and ocular) or patient-tailored analysis of the presented rosacea phenotype. Rosacea etiology and pathophysiology are poorly understood. However, recent findings indicate that genetic and environmental components can trigger rosacea initiation and aggravation by dysregulation of the innate and adaptive immune system. Trigger factors also lead to the release of various mediators such as keratinocytes (for example, cathelicidin, vascular endothelial growth factor, and endothelin-1), endothelial cells (nitric oxide), mast cells (cathelicidin and matrix metalloproteinases), macrophages (interferon-gamma, tumor necrosis factor, matrix metalloproteinases, and interleukin-26), and T helper type 1 (T _H_1) and T _H_17 cells. Additionally, trigger factors can directly communicate to the cutaneous nervous system and, by neurovascular and neuro-immune active neuropeptides, lead to the manifestation of rosacea lesions. Here, we aim to summarize the recent advances that preceded the new rosacea classification and address a symptom-based approach in the management of patients with rosacea.

## From old to new rosacea classification

The first classification of rosacea was published in 2002, recognizing rosacea as a syndrome that is comprehensively depicted by four distinct clinical subtypes defined as erythematotelangiectatic, papulopustular, phymatous, and ocular rosacea ^1^. The classification was the first to systemize rosacea diagnosis in the daily medical routine and gave resilient criteria at hand to efficiently evaluate therapeutic success in each subtype. The implementation of this new tool in the medical daily routine prompted a marked improvement in the medical care of rosacea patients worldwide. In general, the classification suggests a progression from one subtype to another over time and thereby supports a tendency to disregard deviating rosacea manifestations and overlaps between the subtypes. The progress in scientific insight that was accumulated since the introduction of the original rosacea classification in combination with our detailed clinical experience favored a modernized view of rosacea’s pathophysiology as a product of multivariate disease processes that underlie the patient-specific clinical rosacea presentations ^2, 3^. An updated rosacea classification was published in 2016, emphasizing a more patient-centric phenotype approach ^4, 5^. This review aims to summarize the recent developments in our understanding of rosacea’s pathophysiology that preceded the release of the modified rosacea classification and to illustrate the therapeutic management of individual rosacea patients on the basis of their symptoms.

## Epidemiology and diagnosis of rosacea

In the US alone, more than 16 million patients are affected by rosacea, and worldwide incidences peak as high as 18%, particularly in populations with a predominant “Celtic” heritage, such as is observed in Ireland ^6^. Worldwide, the prevalence is estimated to reach over 5%. Females and males are affected equally ^7^. However, the prevalence of rosacea in many countries, including large countries like China and Australia, is still poorly explored, and the prevalence, especially of erythematous rosacea, demands careful differentiation from that of other erythematous diseases and origins of flushing, such as neuro-endocrine tumors.

Rosacea typically arises symmetrically in the central face with gender- and age-specific preferences with regard to lesion qualities. For instance, rhinophyma nearly exclusively presents in the male gender, flushing and erythema often are the first disease signs in younger ages, and telangiectasias make up first rosacea lesions in older ages. The overall rosacea manifestations are flushing, transient or persistent erythema, telangiectasia, papules, pustules, phymata, and (micro)edema ( Table 1) ^1, 8^. Additionally, patients often report stinging or burning pain and very rarely pruritic sensations. Despite the typical centro-facial localization of rosacea, a causative association with the unique centro-facial skin composition that is characterized by a dense presence of sebaceous glands, dense nerval and vascular networks, and *Demodex* mites cannot be drawn conclusively as of yet. However, *Demodex* infestation is increased in some patients with rosacea, and eradication seems to alleviate rosacea symptoms probably by preventing the formation of pro-inflammatory cytokines ^9, 10^.

**Table 1.** Novel classification of rosacea on the basis of diagnostic, major, and secondary features of rosacea.

Diagnostic features	Major features	Secondary features
Persistent centro-facial erythema associated with aggravation by trigger factors	Flushing/transient erythema	Burning sensation
Phymatous changes	Inflammatory papules and pustules	Stinging sensation
	Telangiectasia	Edema
	Ocular manifestations Lid margin telangiectasia Blepharitis, keratitis, conjunctivitis, and sclerokeratitis	Dry sensation of the skin

Adapted from Gallo *et al*. ^2^ and Tan *et al*. ^17^

The patient’s awareness about rosacea’s centro-facial localization often promotes severe psychosocial symptoms, including impaired self-esteem, problems in socializing, and changes in the way the patient thinks, feels, or copes. Recent epidemiological studies confirm these clinical observations and report significant psychological disease burden and decreased quality of life in patients with rosacea ^11– 16^.

Rosacea can be initiated or aggravated by a variety of endogenous and exogenous trigger factors, including heat, noxious cold, ultraviolet (UV) irradiation, and food and beverages. Activation pathways to some of the rosacea triggers have been delineated recently ( Table 2) and might point to future therapeutic targets. The identification of patient-specific trigger factors represents the main and fundamental pillar of rosacea therapy and enables selective and therapeutic trigger avoidance. This strategy in particular is helpful to prevent or alleviate rosacea manifestations that respond dynamically to a trigger such as flushing and transient erythema. In general, the individual clinical manifestation of rosacea appears to be influenced by the special trigger constellation and susceptibilities of a patient. For instance, a bald patient with an enhanced susceptibility toward UV irradiation is imperiled to develop frontoparietal papulopustular lesions, whereas a patient devoid of UV susceptibility is less likely to develop similar rosacea lesions.

**Table 2.** Common rosacea triggers and their activation pathways and current therapy regimen.

Pathway of activation	Trigger	Therapeutic regimen
Inflammasome (NALP3)	Sun exposure, wind, heavy exercise, alcohol consumption, emotional stress, skin care products and cosmetics (formaldehyde), medication, and microorganisms	Avoidance, anti-inflammatory therapy, and antibiotics
TLR-2	Sun exposure, emotional stress, alcohol, exercise, microorganisms/gut microbiome, topicals, and medication	Avoidance, 30+ SPF sunscreen, and brimonidine
TRPV1	Emotional stress, heat/hot weather/hot steam, exercise, alcohol, and spicy food (capsaicin)	Avoidance and brimonidine
TRPV2	Heat	Avoidance
TRPV4	Sun exposure/ultraviolet irradiation, humidity, and osmotic changes	Avoidance and 30+ SPF sunscreen
TRPA1	Cold weather, garlic/mustard oil (pungency), and skin care products and cosmetics (formaldehyde)	Avoidance and brimonidine
PAR _2_	Proteinases and microorganisms	Anti-inflammatory therapy and antibiotics

NALP3, NACHT, LRR, and PYD domain-containing protein 3; PAR _2_, proteinase-activated receptor 2; SPF, sun protection factor; TLR-2, Toll-like receptor 2; TRPA1, transient receptor potential ankyrin 1; TRPV, transient receptor potential vanilloid.

The newly introduced classification of rosacea emphasizes the importance of each rosacea manifestation and distinguishes diagnostic features (signs) from major and secondary features (symptoms) ( Table 1) ^17^. Briefly, phymatous changes and persistent centro-facial erythema are considered the only diagnostic features (signs) of rosacea, whereas flushing, telangiectasia, and inflammatory papules/pustules are considered major symptoms and only in combination can suggest the diagnosis of rosacea. Stinging or burning pain, edema, and dry sensation are defined as secondary features of rosacea ( Table 1).

## Novelties in the pathophysiology of rosacea

Rosacea skin is characterized by dysregulated inflammatory (perivascular or pilosebaceous infiltrate), vascular (dilation), lymphatic (dilation), glandular (hyperplasia), and fibrotic processes, a composition that reflects the multivariate process of the skin disease. Simultaneously, this heterogeneous histological picture hints at rosacea’s unclear pathophysiologic event of onset. Does rosacea originate from an initial dysregulation in inflammatory processes? Is rosacea a disease of the vasculature or rather of the lymphatic system? Does it result from glandular tissue? Or does rosacea represent a skin disease that ultimately arises from combined dysfunctional systems that could involve the gut?

## Immunity

The adaptive immune system along with the innate immune system might take a central part in rosacea’s pathophysiology. Both the early stage perivascular and later-stage pilosebaceous infiltrates are strongly composed of T helper type 1 (T _H_1) and T _H_17 cells and show marked expression of innate immune cells such as additional macrophages and mast cells in papules and erythema and additional neutrophils in pustules and plasma cells in phymata ^18, 19^. CD4 ^+^ T _H_ cells dominate the immune cell infiltrate, but overall rosacea, like its differential diagnosis acne vulgaris, displays a T _H_1/T _H_17 polarization pattern ^20^. These immuno-histochemical findings have been confirmed by transcriptome analysis, where markedly elevated expressions of T _H_1 signature genes—interferon-gamma and tumor necrosis factor-alpha (TNF-α)—and upregulated expression of T _H_17-associated genes coding for IL17A, IL22, IL6, IL20, and CCL20 were found. Neither T _H_2-associated genes nor signature markers for regulatory T cells (FOXP3, IL10, CCR4, and CCR8) were found to be altered in the expression in rosacea when compared with healthy skin ^19^. In acne vulgaris, *Propionibacterium acnes* capably drives the polarization of the immune cell infiltrate to a T _H_1/T _H_17 type ^21, 22^. This mechanism too could be of importance in rosacea, since some studies could associate patient colonization with *Demodex* spp., *Bacillus oleronius*, *Staphylococcus epidermidis*, *Helicobacter pylori*, and *Bartonella quintana* with the development of rosacea ^19, 23– 25^. Rosacea’s association with facial *Demodex* spp. colonization was described quite some time ago, but its consequence for rosacea pathophysiology is still not understood and is even disputed by some. These concerns were substantiated by multiple clinical trials that achieved a reduction or eradication of *Demodex* colonization but often did not observe a marked amelioration of the clinical presentation in patients ^26^. However, in case microbes initiate the shift of the infiltrate observed in rosacea toward a stable T _H_1/T _H_17 polarization, eradication of an “early” rosacea signal might have a smaller therapeutic effect than anticipated in later disease stages. The causative role of especially *Demodex* spp. and *Demodex*-dependent and -independent microbes for the T _H_1/T _H_17 polarization observed in rosacea will need to be investigated, and the importance of the T-cell pattern for the initiation and perpetuation of rosacea is in need of detailed clarification.

Another pathogen that has been suggested to be involved in the pathophysiology of rosacea is *H. pylori* ^27, 28^. However, a recent meta-analysis found only a weak association between *H. pylori* infection and rosacea and between successful eradication of *H. pylori* and improvement of rosacea manifestations ^29^.

## Microbes and the cathelicidin axis in rosacea

**Figure 1.**
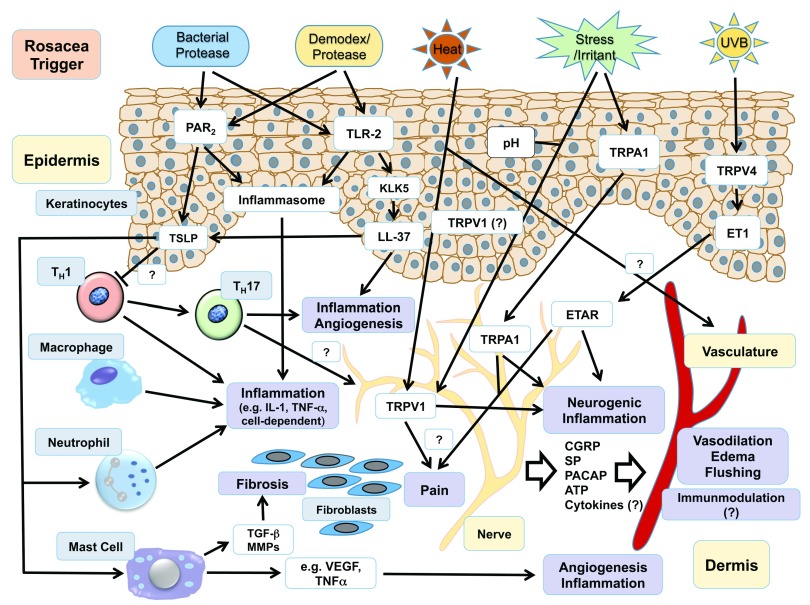
Current understanding of the pathomechanisms in rosacea. Rosacea triggers lead to the activation of downstream effectors (white boxes) in various cell types (gray boxes) probably by the activation of a few specific receptors and channels (white boxes), which in cooperation nurture processes of (neurogenic) inflammation, including edema and vasodilation, fibrosis, pain, and angiogenesis (lilac boxes). For instance, epidermal and probably immune cell-expressed proteinase-activated receptor-2 (PAR _2_) and Toll-like receptor-2 (TLR-2) are activated by rosacea-associated bacterial and *Demodex*-derived proteases, leading to the induction of the inflammasome and subsequent release of pro-inflammatory agents such as tumor necrosis factor-alpha (TNF-α) and interleukin-1 (IL-1) as well as enhanced expression of the innate immune peptide LL-37. ATP, adenosine triphosphate; CGRP, calcitonin gene-related peptide; ET1, endothelin-1; ETAR, endothelin A receptor; KLK-5, kallikrein-5; LL-37, cathelicidin; MMP, matrix metalloproteinase; NALP3, NACHT, LRR, and PYD domain-containing protein 3; PACAP, pituitary adenylate cyclase-activating peptide; SP, substance P; TGF-β, transforming growth factor-beta; TRP, transient receptor potential; TSLP, thymic stromal lymphopoietin; VEGF, vascular endothelial growth factor.

Products of microbes can be recognized by cells of the innate immune system and activate, for example, Toll-like receptors (TLRs) and the G-protein-coupled receptor proteinase-activated receptor 2 (PAR _2_) that are expressed by keratinocytes and can nurture inflammatory processes ^30^. Notably, TLR-2 and probably PAR _2_ are upregulated in patients with rosacea, and *in vitro* activation of both receptors promotes the activation of cathelicidin, an anti-microbial peptide that is also overexpressed in patients with rosacea ( Figure 1) ^30, 31^. TLR-2 signaling can further activate the NLRP3 inflammasome with subsequent IL-1β- and TNF-mediated inflammation amplification and prostaglandin E _2_ synthesis, which support pustule formation, pain sensation, and vascular responses ^32^. TLR-2 activation additionally promotes the release of pro-inflammatory cytokines, chemokines, proteases, and pro-angiogenic factors, which are mediators associated with rosacea symptoms such as erythema, telangiectasia, or inflammation or a combination of these ^10, 33– 35^. A direct link among *Demodex* mites, microbes, and PAR _2_ or TLR-2 has not yet been demonstrated in rosacea. Also, functional *in vivo* data for cathelicidin in humans are still lacking. In mice, TLR-2-induced cathelicidin activation needs functional kallikrein-5 (KLK-5) protease activity for the formation of rosacea-like erythema and angiogenesis ^36^. However, KLK-5 is increased in patients with rosacea and KLK(s)-5 can activate PAR _2_^37^. PAR _2_ is a known mediator of (neuro)inflammation, pruritus and pain sensation, T-cell and neutrophil recruitment to sites of inflammation, mast cell degranulation and vasodilation, and promotion of release of inflammatory mediators such as IL-1, IL-6, IL-8, TNF, chemokines, matrix metalloproteinases, and prostaglandins ^38^. PAR _2_ is expressed by various skin cell types, including keratinocytes, endothelial cells, and innate and adaptive immune cells, activated by microbe-derived proteases, and interacts with TLRs ^39– 43^. The upstream signal or signals of enhanced PAR _2_, TLR-2, and KLK-5 proteins in rosacea have not been identified yet. However, vitamin D can increase TLR-2 and KLK-5 expression in keratinocytes and is found in excess in some patients with rosacea and might be a candidate ( Figure 1) ^44^. Further activation of pro-inflammatory receptors (for example, PAR _2_) can lead to a secondary skin barrier deficit which may lead to more inflammation ^45, 46^.

The importance of microbes and microbe-associated products and their respective target receptor(s) for rosacea pathophysiology is further supported by the recent observation that rosacea patients with SIBO (small intestinal bacterial overgrowth) benefit from treatment with the T-cell modulator rifaximin and present with improved rosacea symptoms ^47– 50^.

## Neurovascular processes and neurogenic inflammation in rosacea

Patients with rosacea react to a vast panel of trigger factors such as temperature changes, heat, cold, exercise, UV radiation, and spicy food and alcoholic beverages with deterioration of rosacea lesions ^51^. The precise mediating receptors and messengers for each trigger factor are in most cases not identified, but recent transcriptomic analysis and immunohistochemical findings indicate that the transient receptor potential family—in particular, members of the ankyrin subfamily (TRPA1) and the vanilloid subfamily (TRPV1 and TRPV4)—might convey cellular responses to several of the rosacea-specific trigger factors ^52, 53^. TRPV1 and TRPA1 are well-described targets for various pungent compounds such as capsaicin (TRPV1) and mustard oil (TRPA1 and TRPV1) ^54^ and could render rosacea stimuli such as heat (TRPV1) ^55^, possibly cold temperatures (TRPA1) ^56^, UVB irradiation (TRPV4) ^57^, and toxins and cosmetics ingredients (for example, TRPA1) ^58^ into clinical rosacea manifestations. In particular, neuronally expressed TRP channels could be responsible for the activation of the cutaneous vasculature leading to flushing, one hallmark feature of rosacea, and erythema by a neurovascular mechanism involving neurogenic inflammation mediators (see Figure 1) ^59– 61^. Rosacea “trigger factor activated TRP channels” in fact lead to the release of vasoactive neuropeptides such as substance P (SP), pituitary adenylate cyclase-activating peptide (PACAP), and the migraine-associated calcitonin gene-related peptide (CGRP) ^61– 63^. Sensory nerves also express the neuro-inflammatory TLR-2 and PAR _2_ which might perpetuate the neurovascular dysregulation observed in rosacea. Because TRP channels (particularly TRPA1 and TRPV1) and PAR _2_ can crosstalk with neuropeptide receptors or at least trigger neuropeptide release, these interactions could help to sustain the neurovascular loop and neurogenic inflammation in rosacea ^52, 53, 64^.

Along sensory nerves, dysregulation of the autonomic nervous system (ANS) can produce facial flushing. However, a distinct role for this mechanism in rosacea-typical flushing is not confirmed at this stage but could be promoted by ANS-expressed PACAP or stress-induced increase of skin sympathetic nerve activity ^65– 67^. In summary, the neurovascular circuits that appear to be involved in rosacea’s pathophysiology might explain the patient-specific trigger profiles and differences in the clinical presentation of rosacea. Altered downstream signaling and target structures might account for the phenotypic variability observed in rosacea.

## Genetics of rosacea

The existence of genetic traits that underlie rosacea is expressed by a positive family history for rosacea. Only recently, a handful of genome-wide association studies have been conducted that propose genetic risk loci for rosacea. An American study included individuals of European descent (all customers of the genetic company 23andme, Mountain View, CA, USA) identified single-nucleotide polymorphisms in the butyrophilin-like 2 (BTNL2) and the human leukocyte antigen–DRA genes. Both genes are associated with the major histocompatibility complex of the acquired immune system, indicating a central role for dysregulations of the immune system in rosacea’s pathogenesis. Another study found a null mutation polymorphism in the glutathione S-transferase (GST) gene that encodes for an enzyme involved in cellular oxidative stress ^68, 69^. In a case study of a patient with granulomatous rosacea, a polymorphism in the NOD2/CARD15 gene was observed ^70^. NOD2/CARD15 protein functions as a caspase recruitment protein and is associated with the function of innate immune system receptors such as TLR-2 and subsequent inflammatory processes. Interestingly, a recent population-based case control study revealed that rosacea shares genetic risk loci with various autoimmune diseases such as multiple sclerosis, type 1 diabetes mellitus, celiac disease, and rheumatoid arthritis. This observation underlines the importance of a thorough risk assessment for the individual patient with rosacea at risk of developing an autoimmune disorder so that multidisciplinary medical care can be organized for affected patients ^71^.

## Therapeutic management of rosacea

### General skin care

Consequent adherence to skin care advice and consequent application of adequate non-irritating skin care can significantly prevent events of rosacea aggravation and improve the patient’s quality of life. Skin care advice consists primarily of avoidance of trigger factors (including stress management), usage of sunscreen with 30+ sun protection factor, application of moisturizers for dry skin and drying applications for oily skin, and gentle cleansing of the whole face ^72^.

## Symptom-based treatment

Because rosacea symptoms derive from distinct pathophysiologies, the therapeutic regimen will in most cases consist of combinations of topicals with systemics or physical therapy or both ^73, 74^.

### Flushing and erythema

According to recent guidelines, two approved topicals can be used to treat persistent erythema in adults with rosacea: brimonidine ^75– 77^ (a beta2-adrenergic agonist) and oxymetazoline hydrochloride 1% cream ^78^ (an alpha1A-adrenoreceptor agonist that also activates alpha2-receptors at higher concentrations) ^4, 17, 72, 73^.

Certain laser therapies can be used, but they should be avoided in pain-sensitized patients ^79– 81^. Off-label usage of beta-blockers such as carvedilol or adrenergic receptor modulators (for example, brimonidine) may alleviate these symptoms ^80^. In case of pain association, an analgesic therapy with, for instance, lidocaine gel (4%) or polidocanol cream in mild cases and antiphlogistics (for example, ibuprofen), anti-depressants (for example, amitriptyline), or anticonvulsants (for example, gabapentin and pregabalin) in more severe cases may be helpful ^82^.

### Telangiectasia

Only a few options exist for the treatment of telangiectasia, among which physical laser therapy and intravascular aethoxysklerol (0.5%–1%) injections are most commonly used.

## Papules and pustules

Patients with mild to moderate papules and pustules benefit from topical treatment with ivermectin (1%) ^83^, metronidazole (1%) ^84– 86^, azelaic acid (15%) ^87^, or sodium sulfacetamide sulfur ^88^. Off-label therapy with topical erythromycin (2%), isotretinoin, clindamycin, permethrin, doxycycline, minocycline, and oral erythromycin has been reported with good results. Combination therapy often helps to prolong symptom-free periods. In severe or therapy-refractory cases, systemic treatment with metronidazole, clarithromycin, and azithromycin can be conducted. In cases of *Demodex* infestation, permethrin (off-label) or ivermectin cream and oral ivermectin can improve the therapeutic result ^72, 89^.

## Phymata

Patients affected by mild phymata benefit from tetracycline therapy. Standard therapies for progressed phymata are ablative (destructive) laser and dermato-surgery. Low-dose isotretinoin appears to reduce phymata by its anti-inflammatory capacity and by lessening the number of sebaceous glands and inhibiting their proliferation ^90– 92^. Systemic immunomodulatory therapy such as dapsone was used in some cases with mixed results.

## Facial (lymph)edema

No US Food and Drug Administration-approved therapy exists. Immunomodulators such as isotretinoin and dapsone or combination therapies with doxycycline/prednisolone have been tried, but the therapeutic value is unclear ^93^.

## Ocular rosacea

The appropriate treatment of ocular rosacea requires a multidisciplinary effort from ophthalmologists and dermatologists. Basic lid hygiene routines such as warm compresses and lubricating drops can be conducted by the patient. Artificial tear substitutes help ocular dryness and accompanying burning and stinging. Successful therapy with topical ivermectin was recently reported ^94^. In more severe cases, cyclosporine eye drops and systemic tetracycline can be prescribed ^73^.

## Summary and future directions

Basic, translational, and clinical research has significantly increased our understanding of a common skin disease, rosacea, leading to novel anti-inflammatory and anti-erythematous treatments. Combination therapies, similar to those for acne and atopic dermatitis, are a key for the successful therapy of this poly-symptomatic disease ^95^.

Several questions remain to be answered: which genes are involved in rosacea? What is the prevalence of rosacea globally? Which are the key mediators and receptors of rosacea in the various clinical symptoms and signs? Which comorbidities are associated with rosacea? How can we optimize the diagnosis and treatment of rosacea? Translational research is demanded to better understand and treat this commonly neglected skin disease.
